# ZIKA virus reveals broad tissue and cell tropism during the first trimester of pregnancy

**DOI:** 10.1038/srep35296

**Published:** 2016-10-19

**Authors:** Hicham El Costa, Jordi Gouilly, Jean-Michel Mansuy, Qian Chen, Claude Levy, Géraldine Cartron, Francisco Veas, Reem Al-Daccak, Jacques Izopet, Nabila Jabrane-Ferrat

**Affiliations:** 1 CPTP, INSERM U1043, CNRS UMR5282, Université Toulouse III, 31024 Toulouse, France; 2 Laboratoire de Virologie, IFB, CHU Toulouse, 31059 Toulouse, France; 3 Service de Gynécologie-Obstétrique, Clinique Sarrus-Teinturiers, 31300 Toulouse, France; 4 Service de Gynécologie-Obstétrique, CHU Toulouse, 31059 Toulouse, France; 5 IRD, UMR-Ministère de la Défense, Faculté de Pharmacie, Université de Montpellier, 34094 Montpellier, France; 6 INSERM UMRS976, Université Paris Diderot, Hôpital Saint-Louis, 75010 Paris, France

## Abstract

The outbreak of the Zika Virus (ZIKV) and its association with fetal abnormalities have raised worldwide concern. However, the cellular tropism and the mechanisms of ZIKV transmission to the fetus during early pregnancy are still largely unknown. Therefore, we *ex vivo* modeled the ZIKV transmission at the maternal-fetal interface using organ culture from first trimester pregnancy samples. Here, we provide evidence that ZIKV strain circulating in Brazil infects and damages tissue architecture of the maternal *decidua basalis*, the fetal placenta and umbilical cord. We also show that ZIKV replicates differentially in a wide range of maternal and fetal cells, including decidual fibroblasts and macrophages, trophoblasts, Hofbauer cells as well as umbilical cord mesenchymal stem cells. The striking cellular tropism of ZIKV and its cytopathic-induced tissue injury during the first trimester of pregnancy could provide an explanation for the irreversible congenital damages.

The Zika virus (ZIKV) is a single strand positive sense RNA virus that belongs to the *Flaviviridae* family. ZIKV infection is mostly asymptomatic and can be associated in some cases with a self-limiting mild illness^1^. However, the recent outbreak of ZIKV has been associated with major fetal abnormalities^1^,^2^.

Murine models have demonstrated that ZIKV infection damages the central nervous system, slows fetal growth and leads to miscarriage^3^,^4^,^5^. However, mouse pregnancy differs from human pregnancy in many ways. The human hemo-monochorial placentation is characterized by a unique fetal trophoblasts invasion into the maternal *decidua basalis* (decidua). Another hallmark is the establishment of immunoprivileged fetal-maternal interfaces where fetal tissues are in close contact with the maternal immune system. The two main interfaces are the decidua that serves as the anchoring point for the placenta and the intervillous space where maternal blood bathes the floating placental villi^6^,^7^. Placental villi are covered by an outer multinuclear syncytiotrophoblast layer (STBs) and an inner mononuclear cytotrophoblast layer (CTBs). At the base of the anchoring villi, proliferative CTBs differentiate into extravillous trophoblasts (EVTs), invade the decidua and remodel uterine arteries to ensure a proper blood flow into the intervillous space^6^,^7^. Thus, viral pathogens that infect the mother can reach the fetus either by hematogenous spread to the placenta or by cellular transfer from the maternal decidua to the anchoring placental villi. Bayer *et al*. have suggested that fetal trophoblasts from full-term pregnancy are resistant to ZIKV infection^8^ while Quicke *et al*. have found that fetal macrophages - Hofbauer cells (HBC) - and to a lesser extend trophoblasts are permissive to ZIKV^9^. Other studies have nonetheless suggested that ZIKV-induced microcephaly is more likely to occur during the first trimester of pregnancy^10^,^11^,^12^. However, the *cellular tropism* and v*irulence* remain, as of yet, unexplored.

The purpose of our study focuses on exploring viral cell tropism using first trimester samples to shed new light on ZIKV pathogenesis. By using an *ex vivo* organ culture model, we demonstrate herein that the maternal decidua, fetal placenta and umbilical cord are permissive to ZIKV infection. Furthermore, the virus targets foundational cell types such as macrophages, fibroblasts, trophoblasts as well as mesenchymal stem cells (MSC). In addition to understanding fundamental mechanisms of ZIKV pathogenesis and its maternal-fetal transmission, our findings pave the way for future strategies to prevent congenital ZIKV infections.

## Results

### Maternal *decidua basalis* supports active ZIKV replication

**Figure 1:**
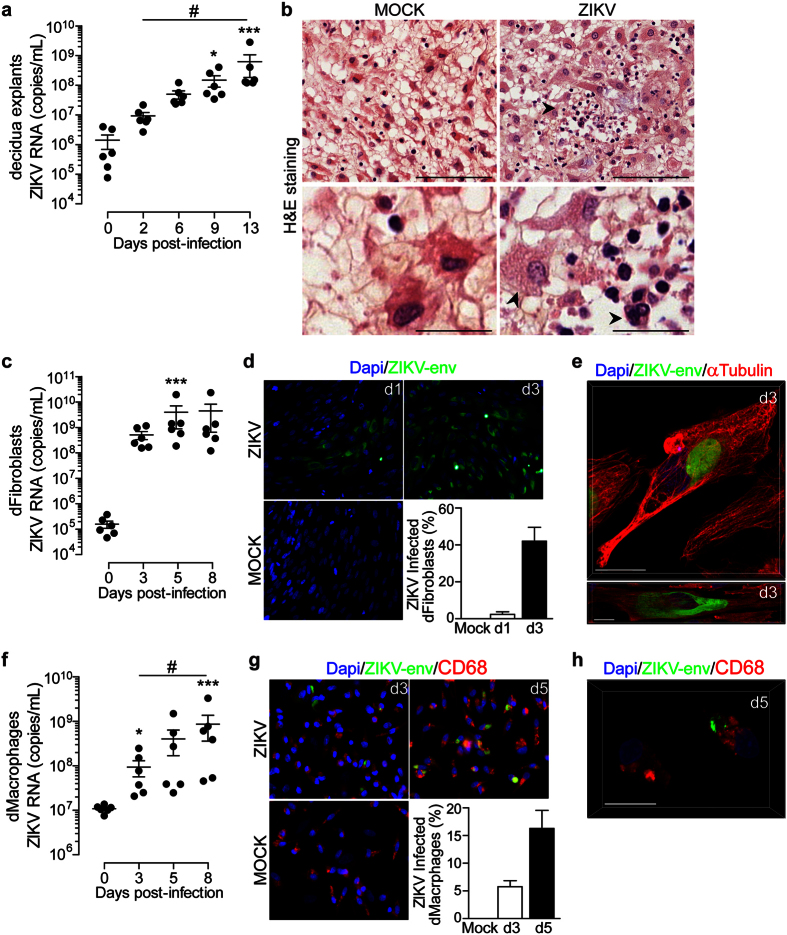
ZIKV replicates in first trimester decidua tissue and cells. (**a**) Decidua explants were infected with 6.10^10^ copies/mL of ZIKV. Viral titer was determined in culture supernatant by qRT-PCR. Data are represented as the mean of three biological replicates for each donor (n = 6). (**b**) Histopathological analysis of H&E-stained sections prepared from mock- and ZIKV-infected deciuda organ. Arrowhead point to ZIKV-induced cytopathic effect (top). Scale bar, 100 μm. Enlarged micrograph shows vacuolar cytoplasm and nuclear changes illustrated by karyorrhexis (bottom, arrowheads). Scale bar, 25 μm. (**c**) Kinetic of ZIKV replication in decidual fibroblasts (dFibroblasts) as determined by qRT-PCR (n = 6). (**d**) Representative large field view of mock- or ZIKV-infected dFibroblasts (ZIKV-env) at day 1 (d1) and day 3 (d3) post-infection. Bar graph represents the mean values determined from ten fields of view for each donor (n = 4). (**e**) 3D reconstitution of confocal microscopy of ZIKV-infected dFibroblasts at d3. Two representative profiles of ZIKV envelope staining. Scale bar, 20 μm. (**f**) Kinetic of ZIKV replication in decidual macrophages (dMacrophages) as determined by qRT-PCR (n = 6). (**g**) Representative large field view of mock- or ZIKV-infected dMacrophages (ZIKV-env) at day 3 (d3) and day 5 (d5) post-infection. Bar graph represents the mean values determined from ten fields of view for each donor. (n = 4). (**h**) 3D reconstitution of confocal microscopy of ZIKV-infected dMacrophages at d5. Scale bar, 20 μm. Primary cells were infected with ZIKV at a MOI of 1. Mean ± s.e.m. of n independent donors are represented. Friedman’s test with Dunn’s multiple comparison post-test was used. (*) represents statistical comparison between day 0 and other time points (#) represents statistical comparison between different time points. (*p < 0.05, ***p < 0.001, ^#^p < 0.05).

To provide insights into early critical events involved in ZIKV transmission, we first examined whether the maternal decidua is prone to infection using an *ex vivo* organ culture. Decidual explants were infected with 6.10^10^ copies/mL and viral replication was monitored over time in culture supernatants by qRT-PCR. As shown in Fig. 1a, viral RNA gradually increased over time with a cumulative production of 2 × 10^2^-fold increase two weeks post-infection (Fig. 1a). This viral replication resulted in infectious virions since conditioned media from infected explants were able to infect HIPEC cells, an extravillous trophoblast cell line (Supplementary Fig. S1). Histological analysis of ZIKV-infected samples five days post-infection displayed evident morphological alterations illustrated by the presence of vacuolar cytoplasm and karyorrhexis (Fig. 1b).

Decidual fibroblasts (dFibroblasts) play a critical role in viral replication and dissemination^13^. We therefore obtained primary dFibroblasts from the adherent cellular fraction of collagenase-digested tissue and infected them at a MOI of 1. ZIKV replication was followed by qRT-PCR in culture supernatant (Fig. 1c). The amounts of viral transcripts were markedly high reaching a plateau of 3 × 10^3^-fold increase within three days of infection. We next analyzed the expression of ZIKV envelope antigen (Fig. 1d). Immunostaining revealed a 20-fold increase in the number of infected dFibroblasts between days one and three post-infection with more than 40% of infected cells. The presence of viral foci and their increase in size over time suggest a cell-to-cell viral spread. Further analyses using confocal microscopy showed either localized perinuclear or diffuse cytoplasmic staining suggesting different stages of infection (Fig. 1e).

Decidual macrophages (dMϕ) are targets for viral replication and share some features with dendritic cells including the expression of DC-SIGN, which is an entry receptor for ZIKV^14^,^15^. To further identify the mode of viral spread within the decidua, we assessed the susceptibility of dMϕ to ZIKV infection. Cells were isolated from collagenase-digested tissue by CD14 positive selection and infected at a MOI of 1. In contrast to dFibroblasts, we observed a gradual increase of ZIKV transcripts in dMϕ over time with an overall 70-fold increase after eight days of infection (Fig. 1f). These findings were further strengthened by the presence of ZIKV envelope antigen in dMϕ, expressing the classical lineage marker CD68 (Fig. 1g). More than 15% of dMϕ were infected five days post-infection with a 3-fold increase of viral antigen expression between days three and five. Furthermore, viral envelope antigen were enclosed within vesicular structures as shown by fine confocal microscopy (Fig. 1h).

Taken together, the differential ZIKV replication in a variety of decidual cell types and its induced tissue injury may favor complications and/or viral dissemination to the fetal placenta during the first trimester of pregnancy.

### ZIKV infection damages first trimester fetal placenta

**Figure 2:**
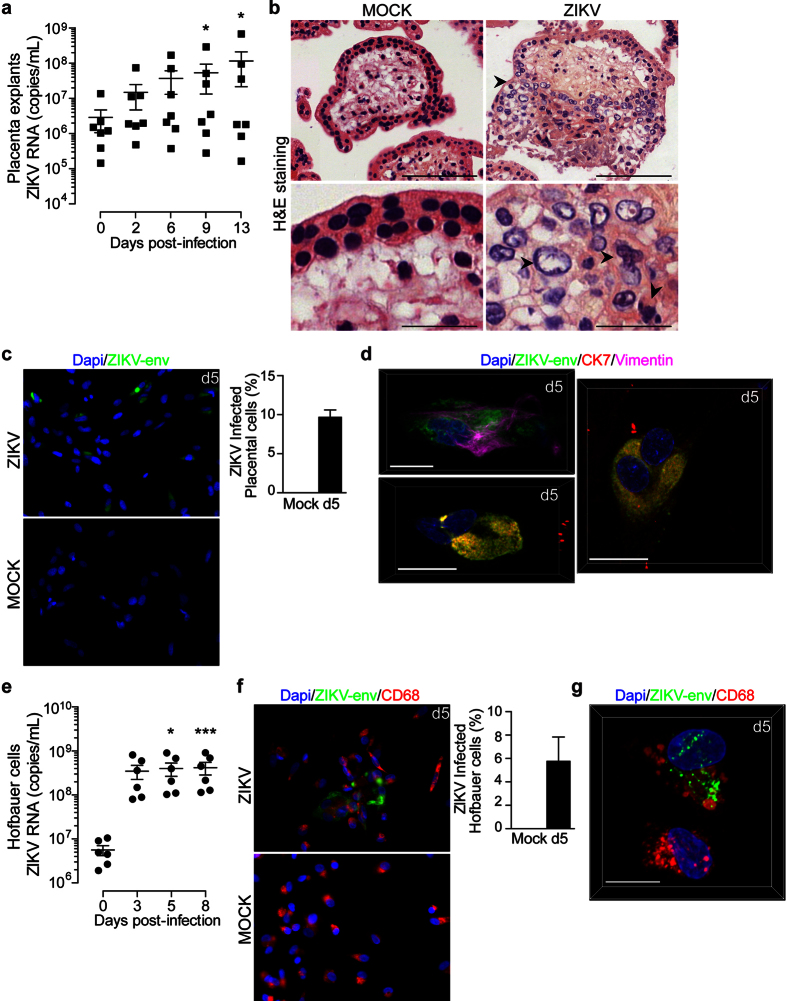
ZIKV replicates in first trimester placental cells and damages tissue architecture. (**a**) Placenta explants were infected with 6.10^10^ copies/mL of ZIKV. Viral titer was determined in culture supernatant by qRT-PCR. Data are represented as the mean of three biological replicates for each donor (n = 7). (**b**) Histopathological analysis of H&E-stained sections prepared from mock- and ZIKV-infected placental organ. Arrowhead point to gross alterations of tissue architecture with changes in trophoblast layers at the surface of the villi (Top). Scale bar, 100 μm. Enlarged micrograph shows nuclear changes illustrated by karyorrhexis and karyolysis (bottom, arrowheads). Scale bar, 25 μm. (**c**) Representative large field view of mock- or ZIKV-infected placental cells (ZIKV-env) at day 5 (d5) post-infection. Bar graph represents the mean values determined from ten fields of view for each donor (n = 4). (**d**) 3D reconstitution of confocal microscopy of ZIKV-infected mesenchymal/fibroblast-like cells (upper left micrograph), CTBs (lower left micrograph) and STBs (right micrograph) at d5. Scale bar, 20 μm. (**e**) Kinetic of ZIKV replication in Hofbauer cells as determined by qRT-PCR. (n = 6). (**f**) Representative large field view of mock- or ZIKV-infected Hofbauer cells (ZIKV-env) at day 5 (d5) post-infection. Bar graph represents the mean values determined from ten fields of view for each donor (n = 4). (**g**) 3D reconstitution of confocal microscopy of ZIKV-infected Hofbauer cells at d5. Scale bar, 20 μm. Primary cells were infected at a MOI of 1. Mean ± s.e.m. of n independent donors are represented. Friedman’s test with Dunn’s multiple comparison post-test was used. (*) represents statistical comparison between day 0 and other time points (*p < 0.05, ***p < 0.001).

Since placental villi are in close contact with the decidua, we assessed their susceptibility to ZIKV infection. To this end, placental explants were infected with ZIKV (6.10^10^ copies/mL) and viral replication was monitored in culture supernatants by qRT-PCR. Kinetic experiments demonstrated that ZIKV is able to infect placental explants (Fig. 2a). However, viral RNA production varied amongst donors. Some placenta displayed a 90-fold increase in viral production over thirteen days of culture whilst others maintained a constant level over time (Fig. 2a). Furthermore, histological analysis of the *ex vivo* mock- or ZIKV-infected organ cultures showed drastic alterations of the placental villi architecture with disruption of the trophoblastic layers (Fig. 2b, top micrograph). Signs of pathogenicity were also identified as major changes in nuclei morphology including karyorrhexis and karyolysis (Fig. 2b, bottom micrograph).

The major cellular component of placental villi are mesenchymal/fibroblast-like cells, trophoblasts and macrophages (HBC). Recent study reported that HBC and to lesser extend trophoblasts from term placenta are permissive to ZIKV^9^. We therefore digested the placental villi in order to define which cells are prone to ZIKV infection during the first trimester of pregnancy. Purified CD14 positive HBC and HBC-negative fraction containing the other placental cells were then infected separately at a MOI of 1. Microscopy analysis revealed that at least 10% of the HBC-negative fraction expressed the envelope antigen five days post-infection (Fig. 2c). Further analysis using confocal microscopy illustrated a perinuclear and a diffused cytoplasmic viral envelope staining within mesenchymal/fibroblast-like cells (Vimentin positive, Fig. 2d upper left micrograph), and in CTBs (CK positive and Vimentin negative, Fig. 2d lower left micrograph). Viral envelope was also found in multinucleated CTBs (Fig. 2d right micrograph) and in CTBs expressing the proliferation marker Ki67 (Supplementary Fig. S2) suggesting that STBs and EVT are also targets for infection. Viral replication kinetics in HBC supernatants displayed an 85-fold increase and reached a plateau three day post-infection (Fig. 2e). Conversely, localized distribution of ZIKV antigen was further observed in 5% of HBC suggesting that the amount of viral RNA does not reflect the percentage of infected cells (Fig. 2f,g).

Collectively, these findings demonstrate that ZIKV can infect the placenta in a donor-dependent manner and disrupt the fetal barrier during the first trimester of pregnancy. Furthermore, the infection of a wide range of cell types in the placental villi can be responsible for the virus spread to the fetus.

### ZIKV replicates extensively in the first trimester umbilical cord

**Figure 3:**
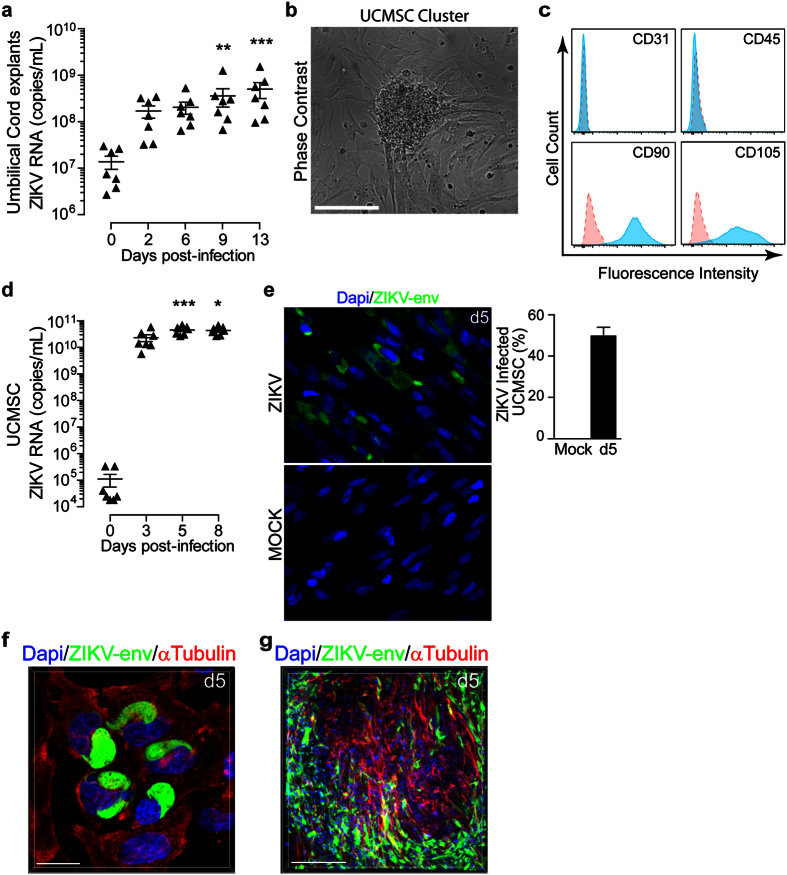
Umbilical cord and mesenchymal stem cells are prone to ZIKV infection. (**a**) Umbilical cord (UC) rings were infected with 6.10^10^ copies/mL ZIKV and viral titer was determined in culture supernatant by qRT-PCR. Data are represented as the mean of three biological replicates for each donor (n = 7). (**b**) Relief phase contrast image of cell cluster and rapidly expanding adherent spindle-shaped fibroblast-like cells compatible with undifferentiated mesenchymal stem cells (MSC) morphology. Scale bar, 200 μm. (**c**) Representative flow cytometry histograms of UCMSC phenotype using CD31, CD45, CD90 and CD105 markers. Specific staining (blue) and control (red). (**d**) Kinetic of ZIKV replication in UCMSC as determined by qRT-PCR (n = 7). (**e**) Representative large field view of mock- or ZIKV-infected UCMSC (ZIKV-env) at day 5 (d5) post-infection. Bar graph represents the mean values determined from ten fields of view for each donor (n = 4). (**f**) 3D reconstitution of confocal microscopy of ZIKV-infected UCMSC at d5. Scale bar, 20 μm. (**g**) 3D reconstitution of confocal microscopy of ZIKV-infected UCMSC-organized cluster at d5. Scale bar, 200 μm. Primary cells were infected with ZIKV at a MOI of 1. Mean ± s.e.m. of n independent donors are represented. Friedman’s test with Dunn’s multiple comparison post-test was used. (*) represents statistical comparison between day 0 and other time points (*p < 0.05, **p < 0.01, ***p < 0.001).

The fact that the first trimester placenta is susceptible to ZIKV infection, along with data showing the presence of the virus in the amniotic fluid^16^ prompted us to investigate whether the virus targets the umbilical cord. Therefore, umbilical cord (UC) rings from first trimester were infected with ZIKV (6.10^10^ copies/mL) and viral RNA was quantified in culture supernatant by qRT-PCR. Results reported in Fig. 3a show a gradual increase of viral genome replication with a cumulative 50-fold increase two weeks post-infection (Fig. 3a).

UC is acknowledged to be a rich source of mesenchymal stem cells (UCMSC). To determine if UCMSC are prone to ZIKV infection, cells were isolated as previously described^17^. After two weeks, UC cultures revealed a monolayer of spindle-shaped fibroblast-like cells as well as cell clusters compatible with undifferentiated UCMSC (Fig. 3b). Flow cytometry analysis showed that more than 95% of UCMSC lack the expression of the endothelial and hematopoietic cell markers, CD31 and CD45. Furthermore, they all express the CD90 and CD105 markers that are characteristic of Wharton’s jelly MSC’s (Fig. 3c).

Next, we infected UCMSC with ZIKV at a MOI of 1 and monitored ZIKV RNA replication over time in culture supernatants. Massive viral replication was observed with a cumulative production of more than a 2 × 10^5^-fold increase three days post-infection (Fig. 3d). Furthermore, 50% of UCMSC were infected and showed a strong perinuclear staining of the viral envelope antigen five days post-infection (Fig. 3e,f). Confocal microscopy also revealed the infection of the UCMSC within the cluster suggesting a cell-to-cell viral spread (Fig. 3g).

Taken together, these data clearly demonstrate that the umbilical cord is highly permissive to ZIKV infection. Moreover, the infection of stem cell progenitors may preclude their multilineage differentiation and may be associated with viral spread to the central nervous system as well as other organs consequently leading to fetus sequelae.

## Discussion

In the wake of the devastating congenital ZIKV infection, we investigated the viral tissue and cell tropism during the first trimester of pregnancy to provide insights into the basis of ZIKV spread to the fetus.

We demonstrate that the maternal decidua, mainly the dFibroblasts and dMϕ are cellular targets for ZIKV infection during the first trimester of pregnancy. These cells provide a favorable microenvironment for successful pregnancy and play a key role in vascular remodeling and tissue homeostasis^7^,^18^,^19^. Thus, the productive ZIKV infection of these fundamental cells would not only favor viral dissemination but may also impair their tissue-support functions^20^. This is further supported by our observation of ZIKV-induced decidua injury. We also found that ZIKV replication in decidual tissue results in infectious virions able to infect an extravillous trophoblast cell line. Collectively, our data suggest that the maternal *decidua basalis* in early pregnancy could serve as a replication platform for ZIKV enabling viral amplification before spread to the placenta through the invasive CTBs at the base of the anchoring villi.

The placental unit is vital for proper fetal development and constitutes an innate barrier to invading pathogens. Our experiment using first trimester placental explants show that ZIKV replication is associated with tissue architecture alteration and could lead to disruption of the placental barrier. This in turn will enable the virus to spread to the fetus. More importantly, some placenta display high viral replication profiles whilst others display low replication profiles. These observations further support the notion that congenital sequelae are not systematic^21^. It is possible that extensive viral replication could lead to placental pathology and fetal infection. Low viral replication on the other hand will result in a self-limiting infection that can be cleared by the local immune system. In fact, the resistance of full-term trophoblasts to ZIKV has been associated with a production of type III interferon^8^ or with the expression of several antiviral genes^9^. Further studies are necessary to define whether similar mechanisms are at play during the first trimester of pregnancy.

Confocal microscopy analyses revealed that ZIKV infects first trimester trophoblasts (CTBs, STBs and EVTs), mesenchymal/fibroblast-like cells and HBC. These cells play a crucial role in both implantation and placentation^22^,^23^. Thus, ZIKV replication might not only hamper their function but also enable the virus to gain access to fetal cells leading to severe complications during pregnancy. Our findings are in agreement with published work showing that term CTBs and HBC are targets for ZIKV^9^. Nonetheless, Bayer *et al*. reported that ZIKV fails to infect syncytialized CTBs from late gestation^8^. Several factors including variability among donors (as describe above), pregnancy stage (early *versus* term) and viral strain may be responsible for these discrepancies. Concomitantly with our work, Tabata *et al*. reported that STBs enclosed ZIKV in small vesicles without an efficient replication. However, ZIKV was detected in CTBs underlying STB layer^24^. Taken together, our work and others suggest that STBs may play a role in ZIKV transmission to villous core even in the absence of viral replication. Thus, viral dissemination route from uterine maternal blood to villous core through the STBs should not be neglected.

The umbilical cord and in particular UCMSC are highly permissive to ZIKV infection. Since trophoblasts are prone to infection, ZIKV might access to UC through fetal blood or amniotic fluid that bathes the UC. Given the fact that MSC can differentiate into different tissues (including the osteogenic, adipogenic and neuronal progenitors^25^), the microcephaly and the neural sequelae observed in congenital ZIKV infections might be explained at least partially by an abnormal differentiation of infected stem/progenitor cells.

Finally, we observed two major profile of viral subcellular localization dependent on cell types. In dMϕ and HBC, the virus was enclosed in cytoplasmic vacuolar-like structures while in the other primary cells the viral envelope was either diffused in the cytoplasm or around the nucleus. RNA viruses including Flaviviruses are known to involve several interaction between viral and host factors in order to build their own replication factories^20^,^26^. Thus, the observed viral localization may be related to different organization of host cell compartments and/or cytoskeleton dynamics that are cell specific. Although we did not characterize the exact architecture of these structures, high viral replication dynamic was observed in cells showing perinuclear and diffused cytoplasmic staining. Whilst a much slower dynamic was seen in cells displaying compartmentalized vacuolar-like viral structures. These observation are in agreement with previous studies where cellular compartment localization were associated to viral replication dynamics^27^. Furthermore, the abundance of host factors, cell cycle progression and cell specific metabolism may impact the efficiency of replication^28^,^29^. Therefore, understanding how ZIKV replication subverts the cellular machinery in different permissive cells is critical to define optimal pharmacologic intervention and reach viral eradication.

In summary, our present work on first-term pregnancy, provide evidence that ZIKV targets a set of tissues critical for fetus development including the maternal decidua, the fetal placenta as well as the umbilical cord. Furthermore, we show that ZIKV replicates differentially in a wide range of maternal and fetal cells, including dFibroblasts, dMϕ, trophoblasts and HBC suggesting that the virus can reach the fetus through a succession of adjacent target cells from the *decidua basalis* to the anchoring villi. Our findings provide groundwork for translational research initiatives and can be used to develop efficient strategies to prevent congenital ZIKV.

## Methods

### Ethics statement

All the patients included in the study have provided written informed consent in accordance with the Declaration of Helsinki guidelines. The study was approved by the Research Ethical Comity Haute-Garonne and Agence de Biomédecine (PFS08-022). All experiments were performed in accordance with the approved guidelines.

### Preparation of organ cultures and cell purification

First-trimester pregnancy samples (7–12 weeks of pregnancy) were obtained from healthy women undergoing vaginal elective termination of pregnancy (a cohort of 26 donors with an age range 18–30 years) as previously described^30^. Briefly, 0.3 cm^2^ tissue explant pieces were prepared from decidua basalis and placenta. Umbilical cord was cut into 2 mm rings. Tissue explants can be maintained up to three weeks in DMEM:F12 (v:v) culture medium with 10% fetal bovine serum (FBS)^15^. Primary cell suspensions were isolated from minced tissues subjected to collagenase IV (Sigma-Aldrich, France) digestion for 45 min at 37 °C under gentle stirring followed by Ficoll-Hypaque density gradient (Amersham Biotech) separation^30^. Decidual macrophages and Hofbauer cells were purified by CD14 positive selection kit according to the manufacturer procedure (Miltenyi Biotec, France). Cell purity was at least 90% as determined by CD14 and CD68 staining. Decidual fibroblasts were purified from the decidual adherent mononuclear cell fraction by successive rounds of mild trypsin treatment^13^. UCMSC were prepared as described elsewhere^17^. Briefly, UC was firstly cut into small segments and cultured in 60 cm^2^ Petri dishes (Corning, USA). Fragments of UC were left undisturbed in culture and monitored for up to two weeks to allow identification of MSC in the dishes. UCMSC at the P1 passage were used in all experiments.

### ZIKV isolation and propagation

The Zika virus used in this study was isolated in January 2016 from semen collected from an infected symptomatic French traveler returning from Brazil^31^. High titer stocks were obtained by passaging the virus in Cercopithecus aethiops, the African green monkey kidney epithelial cell line (Vero). Vero cells were inoculated with seminal fluid for 2 h (1:10 dilution) in DPBS containing 2% BSA. The viral inoculum was removed and cells were cultured in DMEM medium supplemented with 10% FBS. Medium was changed 24 h post viral inoculation. Supernatant was collected from infected cells five days post-infection. Viral stocks were stored in single use aliquots at −80 °C for subsequent infections.

### *Ex vivo* infection of tissue explants and primary cells

Tissue explants and primary cells were infected overnight with 6 × 10^10^ RNA copies/mL (equivalent to a MOI of 0.5) or at a MOI of 1 respectively in DMEM:F12 containing 10% FBS. After several washes in DPBS, tissue explants were cultured in FBS-medium. Decidual explants were cultured on collagen sponge gels as described previously^15^. All tissue explant’s experiments were performed in triplicate for each donor.

### Quantitative ZIKV RT-PCR

RNA was extracted from patient samples and culture supernatants using MagNA Pure 96 DNA and Viral RNA Small Volume kits^®^ on a MagNA Pure 96TM instrument (Roche, France). ZIKV RNA was detected by amplification of an NS5 fragment (Real Star Zika virus RT-PCR kit 1.0, Altona Diagnostic GmbH, Germany) using a Light Cycler 480 instrument (Roche Molecular Systems). The limit of detection was 150 copies/mL. Both assays were performed according to the manufacturer’s instructions. Internal standards were used to correct for potential variation in the amount of input material. Quantitative RT-PCR values were further normalized and are given as viral RNA copy numbers/mL. Viral stocks were titrated using plaque-forming assay. Kinetic of viral production was determined as RNA copy number per mL, calculated based on a standard curve.

### Fluorescent microscopy

Infected cells were fixed with 4% paraformaldehyde, permeabilized with 0.3% Triton X-100 and stained with antibodies against Flavivirus group antigen (D1-4G2-4-15, 1:500 dilution, Merck-Millipore), the classical macrophage marker CD68 (1:100 dilution, Dako), the trophoblast marker Cytokeratin 7 (CK7, 1:100 dilution, Dako), the proliferation marker Ki67 (1:250 dilution, Vector), Vimentin (1:100 dilution, Cell Signaling) and the microtubule marker alpha-tubulin (1:500 dilution, Sigma). Antigen staining was visualized with Alexa Fluor-conjugated class specific secondary antibodies (1:500 dilution, Invitrogen). Nuclei were visualized with 4,6-diamidino-2-phenylindole (Dapi, 1:10,000 dilution, Sigma). Large field microscopy was performed with Leica DM4000B microscope (Leica, Solms, Germany). Confocal z-stacks were captured using LSM710 confocal microscope (Carl Zeiss, Germany). 10×, 20× or 63× oil objectives were used for all acquisitions. Images were processed using Imaris software (Bitplane AG, Switzerland). The quantification values were obtained by calculating the percentage of infected cells over total cells (1,500 cells were analyzed for each donor).

### Histological analysis

Tissue organ cultures were either mock or ZIKV-infected for five days as described above. Tissues were fixed with 10% formalin, embedded in paraffin, and then sliced into 3 μm-thick sections. Thin sections were processed for histology and stained with hematoxylin and eosin (H&E). Photographs were taken with a Leica DMR microscope (Leica Microsystems, Nanterre, France) at ×40 objective, using 3DHISTECH panoramic slide viewer (3DHISTECH Kft, Budapest, Hungary).

### Flow cytometry

Mouse fluorochrome-conjugated anti-human monoclonal antibodies were used. Anti-CD31 (phycoerythrin, 1:50 dilution, BD Pharmingen), anti-CD90 (fluorescein isothiocyanate, 1:50 dilution, BD Pharmingen), anti-CD45 (Vio-blue, 1:50 dilution, Miltenyi), anti-CD105 (allophycocyanin, 1:50 dilution, BD Pharmingen).

Cells were extensively washed in PBS and immunostained at 4 °C using fluochrome-coupled monoclonal antibodies diluted in FACS buffer (PBS, 1% FBS). Analyses were performed on a BD LSR-FORTESSA cytometer. Data were further analyzed using FlowJoTM software 10.1.

### Statistical Analysis

Statistical analyses were carried out with GraphPadTM Prism software version 5.0 f. Friedman test with Dunn’s multiple comparison test was used. Lines represent the mean, and error bars indicate s.e.m. The figure legends show in parentheses the number of independent donors used in each experiment. p values < 0.05 were considered significant. (*) represents statistical comparison between day 0 and other time points (*p < 0.05, **p < 0.01, ***p < 0.001). (#) Represents statistical comparison between different time points (^#^p < 0.05).

## Additional Information

**How to cite this article**: El Costa, H. *et al*. ZIKA virus reveals broad tissue and cell tropism during the first trimester of pregnancy. *Sci. Rep.* **6**, 35296; doi: 10.1038/srep35296 (2016).

## Supplementary Material

Supplementary Information
