# Supplementary material for: ZIKA virus reveals broad tissue and cell tropism during the first trimester of pregnancy
Source: Sci Rep. 2016 Oct 19;6:35296. doi: 10.1038/srep35296 (PMC5069472; doi:10.1038/srep35296)
Supplement: Supplementary Information [file srep35296-s1.pdf]

ZIKA virus reveals broad tissue and cell tropism during the first trimester of pregnancy

Hicham El Costa[1,2,7], Jordi Gouilly[1,7], Jean-Michel Mansuy[2], Qian Chen[1], Claude Levy[3], Géraldine Cartron[4], Francisco Veas[5], Reem Al-Daccak[6], Jacques Izopet[1,2] and Nabila Jabrane-Ferrat[1,*]

[1]*CPTP, INSERM U1043, CNRS UMR5282, Université Toulouse III, 31024 Toulouse, France.*

[2]*Laboratoire de Virologie, IFB, CHU Toulouse, 31059 Toulouse, France.*

[3]*Service de Gynécologie-Obstétrique, Clinique Sarrus-Teinturiers, 31300 Toulouse, France.*

[4]*Service de Gynécologie-Obstétrique, CHU Toulouse, 31059 Toulouse, France.*

[5]*IRD, UMR-Ministère de la Défense, Faculté de Pharmacie, Université de Montpellier, 34094 Montpellier, France.*

[6]*INSERM UMRS976, Université Paris Diderot, Hôpital Saint-Louis, 75010 Paris, France.*

[7]*Co-first author*

[*]Corresponding author: Nabila Jabrane-Ferrat, PhD

E-mail:nabila.jabrane-ferrat@inserm.fr

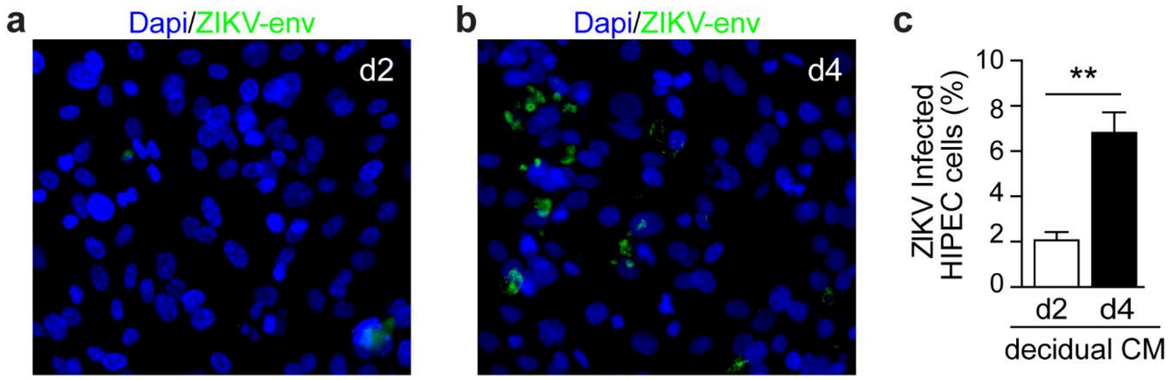

Supplementary Figure S1. **Viral replication in *decidua basalis* explants produce infectious virions**

HIPEC, an extravillous trophoblast cell line, was incubated with day 2 (d2) and day 4 (d4) conditioned media (CM) from infected decidual explants. The presence of infectious virions was monitored by immunostaining of the viral envelope after three days of culture.

(a) Representative large field view of ZIKV-infected HIPEC cultured in CM from d2.

(b) Representative large field view of ZIKV-infected HIPEC cultured in CM from d4.

(c) Bar graph represents the mean ± s.e.m values determined from ten fields of view from six independent experiments. **p=0.0025 using Mann-Whitney test.

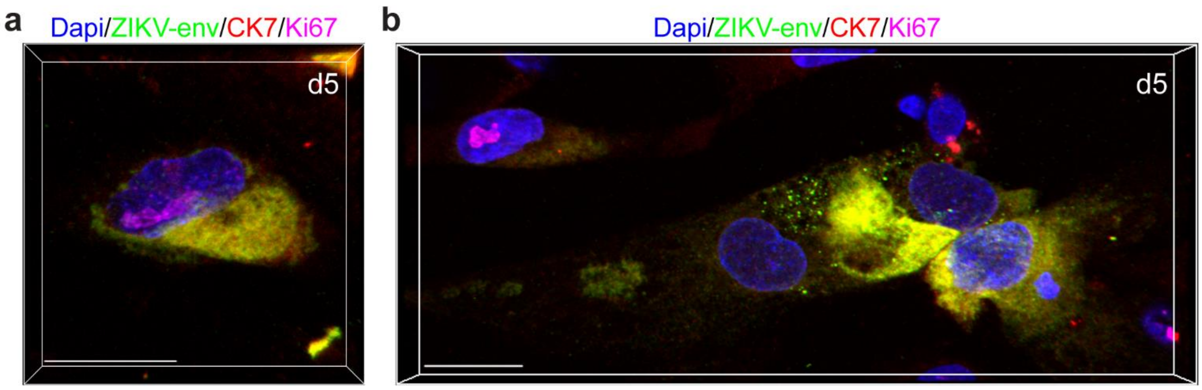

Supplementary Figure S2. **ZIKV replicates in EVTs, CTBs and STBs**
Placental cells were infected with ZIKV at a MOI of 1.
(a) 3D reconstitution of confocal microscopy of proliferating ZIKV-infected EVT at day 5 post-infection (d5). Scale bar, 20µm.
(b) 3D reconstitution of confocal microscopy of ZIKV-infected EVTs, CTBs and STBs at d5. Scale bar, 20µm.
